# Comments upon Recent Obstetrical Literature

**Published:** 1897-03

**Authors:** Jos. Eve Allen

**Affiliations:** Augusta, Ga.


### *MEDICAL PROGRESS.*

---

### Comments upon Recent Obstetrical Literature.

By Jos. Eve Allen, M.D., Augusta, Ga.

The progress of Obstetrics has been so great during the last decade, and new books upon the subject are coming from the press with such confusing rapidity, that the busy practitioner is generally at a loss to select the one that most fully represents the present state of the science. A review, therefore, of the new "Obstetrics" that are accessible to the medical man who reads English only with especial reference to his needs in practice will possibly be of some interest.

The last work that has been published in this country is "A Treatise on Obstetrics," by Professor Edward P. Davis, of Jefferson Medical College, Philadelphia. It is a volume of 540 pages, profusely illustrated, and treats not only of midwifery, but also of diseases of the new-born infant. It is written in a very lucid style, and is evidently the product of close study and wide experience, and is just such a work as would be naturally expected from a man living in a great medical center with vast opportunities for observation and practice.

As an exposition of the physiology and pathology of pregnancy, parturition, and the puerperal state, this work is thoroughly up to date, but as to treatment, some of its teachings are not altogether in accord with Southern practice; for instance, with us the obstetric position is the dorsal decubitus, and the author does not give any valid reason for its abandonment for the English position which he advocates. Again, in the use of quinine the doses advised are much smaller than those we are accustomed to give. The experience of physicians in malarial sections will sustain the opinion that quinine may be given to pregnant women with impunity, and that it has no effect in producing abortion, but will, on the other hand, prevent the occurrence of this accident; and that in those cases of fevers and other affections in which premature delivery has taken place during the administration of quinine, the abortion was not due to the use of the drug, but because it was not given in sufficient quantity. Women who are pregnant require larger doses of quinine than under any other circumstances, and it is generally useless to give less than ten grains at a dose to secure its tonic effect during labor. This drug certainly does exert a tonic and stimulant action upon the hypertrophied muscular fibres of the uterus at term, but has no effect whatever upon the undeveloped tissue of this organ during the earlier months of gestation.

The views of Professor Davis as to the necessity of a thorough and careful examination of every pregnant woman by the accoucheur previous to the beginning of labor are eminently correct, and this should be the routine practice of every man who engages in this line of professional work, for in this way only can complications be foreseen and prevented and the best interest of both mother and child secured. This examination should be made during the seventh, eighth, and ninth months. All of the functions of the economy should be closely investigated, and by palpation, auscultation, and internal and external pelvimetry, the capacity and shape of the pelvic canal and the presentation, and, if possible, the position of the fetus determined. It is to these frequent preliminary examinations that the remarkable results obtained in lying-in hospitals are to be largely attributed.

The chapter on the pathology of pregnancy is particularly good, and includes not only the common, but also many of the uncommon, disorders not usually considered in text-books on obstetrics. The directions for unrinalysis are very full, but entirely too complicated for general practice. The author is right in insisting upon these analyses being made at intervals of two weeks during the latter months, and also in calling particular attention to the fact that it is the amount of urea present in the urine, and not the percentage of albumen, that is of especial clinical interest. It is the amount of urea that infallibly indicates danger, because many conditions may arise during pregnancy that will produce albumenous urine without serious results following, but the normal percentage of urea being two or three per cent., whenever this falls below one per cent. it indicates a state of toxemia that urgently calls for relief. Serious kidney lesions often exist before the chemical composition of the urine becomes materially altered, and hence in all cases a microscopic examination of this fluid should also be made.

A close study of the section on the nausea and vomiting of pregnancy will greatly facilitate the treatment of this perplexing class of cases. It is now clearly demonstrated that with the majority of these patients the use of drugs by the stomach is valuable time lost, and that what is required is proper feeding and local treatment of the pelvic organs. When great prostration and rapidly-increasing anemia is present, death will certainly ensue unless the obstetrician acts promptly and at once terminates the pregnancy. It is wonderful how quickly the emptying of the uterus is followed by arrest of the vomiting; and this operation should always be performed before the woman's reserve force is all exhausted and she gets into a profound depression from which it is impossible for her to react. The mistake usually made in such cases is that the operation is done too late.

The physiology and mechanism of labor is considered in entirely too cursory a manner in view of the importance of the subject.

In works on obstetrics, that portion which treats of the management of natural labor is always the most interesting, for here is to be found the clearest reflex of the author's views and personal experience, from which may often be gained important practical hints and suggestions. Professor Davis does not disappoint us in this regard. His remarks on the management of normal labor, although presenting nothing new, are most judicious and worthy of attention. During the first stage the importance of sustaining the woman, mentally and physically, is urged. Hydrate of chloral is given to secure sleep and moderate the severity of the pains. Antiseptic vaginal douches are not mentioned, and hence the inference is that they are not used; which is very proper, because the micro-organisms found in the vagina under normal conditions are innocuous, and antiseptics before delivery only alter the secretions and interfere with the dilatation of the parts. Ante-partum douching of the vagina is only necessary when this canal is the seat of infection. Examinations of the genital tract should always be made aseptically, and as seldom as is consistent with a proper knowledge of the progress of the case. The obstetrician should always know beforehand the capacity of the pelvis and the fetal presentation, and at the time of labor make vaginal examinations only to determine the stage which has been reached, the condition of the lower uterine segment, the amniotic sac, vulva, etc. In the conduct of parturition the obstetrician should always realize that this is a physiological process that does not often demand the assistance of art, and that his position is generally one of masterly inactivity. He should be prepared to meet promptly any emergency or accident that may occur, but must not interfere as long as the natural forces are adequate to effect the delivery. In this connection the advice of the late Professor Fordyce Barker may be followed with advantage by young practitioners, as it will enable them to keep their heads in the face of danger and maintain that calm demeanor which is so essential to inspire the confidence and co-operation of others. He says: "When summoned to a case of labor, revolve in your mind all the accidents that are likely to happen and settle definitely your line of treatment, so that, should occasion arise, you will be fully prepared to act promptly and properly."

As said before, we do not agree with Professor Davis as to the proper position for delivery. The dorsal position gives the woman better control over her voluntary muscles. In it necessary manipulations, douching, instruments, etc., may be employed with greater facility and the risk of perineal laceration is not increased. Winckel has pointed out that in the lateral decubitus there is danger of the uterine obliquity produced interfering with the engagement of the head and its movements of flexion and rotation.

The anesthetic advised is chloroform as being more manageable than ether and less liable to affect injuriously the kidneys and lungs. The hypertrophy of the left ventricle that takes place during pregnancy makes chloroform usually safe during labor. Before administering it, however, the heart should always be examined, for sometimes in cases of dilatation the most alarming symptoms have been observed to follow moderate inhalation. Chloroform should also be pushed with the greatest caution, for it predisposes to uterine relaxation and consequent post-partum hemorrhage.

The picture on page 195 does not convey any exact idea as to the action of the hands in preventing rupture of the perineum. In the proper performance of this maneuver the upper or pubic hand guages the advance of the head, and by direct pressure upon it during a pain prevents its sudden exit from the vulva. The lower or sacral hand makes upward pressure with the ulnar border upon the structures between the coccyx and rectum thus relaxing the anterior perineum and causing the occiput to hug the pubic arch.

The author's directions for the delivery of the placenta do not accord with the teachings of Crede. The rules given for preparing the woman for bed after delivery are plain and excellent, and the binder is not, as is now fashionable with some obstetricans, condemned, though no great stress is laid upon its employment. The binder is a most valuable appliance and should always be used, tending, as it does, to ensure the safety and adding greatly to the comfort of the lying-in woman. It must be put on under the direct supervision of the physician himself and not left to the hands of friends and nurses, for if so, it will not be properly done, and upon its proper application depends its efficiency. The binder should be pinned evenly and moderately tight from the trochanters to the ensiform cartilage. It thus supports the relaxed abdominal walls and prevents relaxation and misplacement of the uterus, and at the same time guards against those circulatory disturbances which are incident to the sudden decrease of intra-abdominal pressure that occurs after delivery. The binder, by keeping the womb firmly contracted, prevents the accumulation of clots that would give rise to after-pains, favor post-partum hemorrhage, or become the source of infection. When worn during the puerperium it assists greatly the process of involution.

It is impossible in the limited space of a journal article to refer to all of the many good points in Professor Davis's work. Suffice it to say, that it is a valuable contribution to medical literature and furnishes a most reliable guide to the practice of modern obstetrics.

Grandin and Jarman's Practical Obstetrics is an able exposition of advanced thought in this department. The tendency in midwifery at present is more and more toward the adoption of surgical methods of treatment.

A distinguished obstetrician of New York has recently seen fit to express his disapproval of this, but nevertheless it is a great step in the right direction, for it is certain that the nearer obstetric procedures are conducted according to the principles that govern in surgery the better are the results.

In this work surgical methods are fully set forth, and it is shown that the brilliant results of modern obstetrics are the direct outcome of accurate diagnosis and strict asepsis. Pelvimetry and other means of diagnosis are considered at great length, and every detail of asepsis and antisepsis is discussed, and in no other book can fuller information be obtained on these subjects.

The same danger of infection threatens the parturient woman that is present in operative surgical cases and can only be prevented by a rigid adherence to cleanliness in technique. The obstetrician must always remember that success, not only in major operations, but in the simplest service he is called upon to perform, depends upon three factors: (1) A clean field of operation—the genital tract of the patient must be properly prepared. (2) A clean operator—his hands and instruments must be aseptic. (3) A clean environment—the nurse, the bed, the clothing, etc., must be thoroughly aseptic. By this, and this alone, can security from septicemia be made absolutely certain.

The position for delivery recommended offers many advantages over all others, and should be generally adopted. It is the dorsal, with the woman across the bed, her hips brought over the edge, and her feet supported by two assistants. This position allows the accoucheur perfect use of both hands and does not necessitate the moving of the patient should instrumental or other assistance be required.

The views of these writers about the forceps is altogether different from those held by other authorities. They regard this instrument only as a tractor and advise against its use as a lever or compresser. These powers of the forceps are, however, unquestionably important in some cases, but it is best for a beginner to confine himself to direct traction, and not until he acquires skill should he attempt pendulum movements, because bungling efforts at leverage often do great damage to the maternal structures. On the lever power of the forceps Dr. Robert Barnes very properly says: "I believe that pure traction is almost impossible, and I am equally certain that a gentle and careful leverage will enable you to deliver with a great economy of force and time, which means, of course, greater safety to the mother."

Hunter's forceps is the one most prominently figured in the plates. This is an instrument which consists essentially of only blades and lock, and with it only traction can be made. It is, hence, a weak instrument, and being weak is, therefore, dangerous, because it cannot be worked without an undue expenditure of muscular force, and the operator does not have over it that well balanced control which is so necessary to prevent injury.

The work of Professors Grandin and Jarman, is deeply interesting and highly scientific, and should be in the library of every physician who wishes to keep abreast of the times.

